# A Translational Study of TNF-Alpha Antagonists as an Adjunctive Therapy for Preventing Hemophilic Arthropathy

**DOI:** 10.3390/jcm9010075

**Published:** 2019-12-27

**Authors:** Feixu Zhang, Mengyang Xu, Qin Yang, Baolai Hua, Binglan Xia, Zhenyang Lin, Xiao Xiao, Paul E. Monahan, Junjiang Sun

**Affiliations:** 1 School of Bioengineering, East China University of Science and Technology, Shanghai‎ 200237, China; zhangfeixu2009@126.com; 2 School of Pharmacy, East China University of Science and Technology, Shanghai 200237, China; reigetsu@163.com (Z.L.); Xiaoxiao@ecust.edu.cn (X.X.); 3 Department of Hematology, Clinical Medical College, Yangzhou University, Yangzhou 225001, China; xumengyang2019@hotmail.com (M.X.); 18010618742@163.com (Q.Y.); yzsbxbl@163.com (B.X.); 4 Gene Therapy Center, University of North Carolina, Chapel Hill, NC 27599, USA; paul.monahan@sparktx.com; 5 Harold R. Roberts Comprehensive Hemophilia Diagnosis and Treatment Center, University of North Carolina, Chapel Hill, NC 27599, USA; 6 Spark Therapeutics, 3737 Market Street, Philadelphia, PA 19104, USA; 7 Division of Molecular Pharmaceutics, Eshelman School of Pharmacy, University of North Carolina, Chapel Hill, NC 27599, USA

**Keywords:** hemophilia, hemarthrosis, hemophilic arthropathy, TNFα, anti-TNFα

## Abstract

Repeated intra-articular hemorrhages lead to hemophilic arthropathy in severe hemophilia. Inflammation and pro-inflammatory cytokines (e.g., tumor necrosis factor alpha (TNFα)) might be involved in this pathogenesis. We hypothesized that anti-TNFα may provide adjuvant protection for hemophilic arthropathy management. We measured TNFα in synovial lavage from hemophilia mice subjected to hemarthrosis induction and synovial fluid from patients with hemophilic arthropathy (*n* = 5). In hemophilia mice, recurrent hemarthroses were induced, anti-TNFα was initiated either from day (D)7 after one hemarthrosis episode or D21 after three hemarthroses episodes (*n* ≥ 7/treatment group). In patients with hemophilic arthropathy (16 patients with 17 affected joints), a single dose of anti-TNFα was administered intra-articularly. Efficacy, characterized by synovial membrane thickness and vascularity, was determined. Elevated TNFα in synovial lavage was found in the hemophilia mice and patients with hemophilic arthropathy. Hemophilia mice subjected to three hemarthroses developed severe synovitis (Synovitis score of 6.0 ± 1.6). Factor IX (FIX) replacement alone partially improved the pathological changes (Synovitis score of 4.2 ± 0.8). However, anti-TNFα treatment initiated at D7, not D21, significantly provided protection (Synovitis score of 1.8 ± 0.9 vs. 3.9 ± 0.3). In patients with hemophilic arthropathy, intra-articular anti-TNFα significantly decreased synovial thickness and vascularity during the observed period from D7 to D30. Collectively, this preliminary study seems to indicate that TNFα may be associated with the pathogenicity of hemophilic arthropathy and anti-TNFα could provide adjuvant protection against hemophilic arthropathy. Further studies are required to confirm the preliminary results shown in this study.

## 1. Introduction

Bleeding into the joints represents the major morbidity of severe hemophilia. The management of hemophilic arthropathy remains a major concern, especially in undeveloped countries [1,2]. As current prophylactic regimens do not completely prevent joint bleeding, some patients may still develop joint disease [3,4]. Blood in the joint creates an inflammatory cytokine environment with many mediators, such as tumor necrosis factor alpha (TNFα), interleukin (IL)-1, *matrix metalloproteinases*, and others, that are also implicated in the pathology of rheumatoid arthritis (RA) and osteoarthritis [5,6,7]. Previously, we found that FVIII replacement at hemorrhage and anti-IL-6R in FVIII^−/−^ mice decreased joint damage as revealed by the decrease in synovial hyperplasia, hemosiderin deposition, and macrophage infiltration [8] compared to that after treatment with FVIII alone.

The role of TNF-α in the pathogenesis of hemophilia arthropathy has been studied in hemophilia A mouse model. A significant TNFα accumulation was found in the hemorrhagic tissues of the injured knee and strong TNF-α gene upregulation observed since day 3 up to 30 days after hemarthroses. Furthermore, genetic inactivation of TNFα reduced the osteopenia and synovial inflammation that developed in this hemophilic arthropathy mouse model [9]. Nevertheless, in an in vitro study using human cartilage culture, blocking IL-1β, not TNFα, protected blood-induced cartilage damage [10].

Hemophilic arthropathy shares similar pathological changes with RA and the efficacy of anti-TNFα has been extensively used. To further explore the role of TNFα in the pathogenicity and management of hemophilic arthropathy, we hypothesized that in addition to supporting hemostasis with factor replacement, TNFα inhibition as a co-therapy could help to prevent the inflammatory sequelae of hemarthroses. First, we determined the efficacy of TNFα inhibition as an adjunctive therapy in the protection against joint damage after recurrent intra-articular hemorrhage in a hemophilia B mouse model. Given the more localized inflammation in synovium in animal models and patients with hemophilic arthropathy, to avoid the systemic adverse effects of anti-TNFα [11], we translated the in vivo finding to patients with hemophilic arthropathy by delivering TNFα antagonists intra-articularly. We found that a single dose of anti-TNFα via the intra-articular route decreased synovial membrane thickness and vascularity.

## 2. Material and Methods

### 2.1. Animal Care and Study

Factor IX knockout C57Bl/6J (FIX^−/−^) mice were bred in-house. Hemarthrosis induction was performed and tissues were processed as described previously [12,13]. All blood samples were collected from the retro-orbital plexus into 1:9 parts 3.2% citrated sodium. Plasma was collected and stored at −80 °C. All investigations were approved by the UNC-CH Institutional Animal Care and Use Committee.

#### 2.1.1. Drugs Used in the Study

Recombinant human factor IX (FIX, BeneFIX) was purchased from Pfizer (Philadelphia, PA, USA). Anti-TNFα (etanercept) was procured from Amgen (Thousand Oaks, CA, USA). Dexamethasone (Dex) was obtained from Sicor Pharmaceuticals (Irvine, CA, USA).

#### 2.1.2. In Vivo Efficacy of Anti-TNFα in Protecting against Multiple Bleeding-Induced Joint Deterioration in FIX^−/−^ Mice

**Figure 1:**
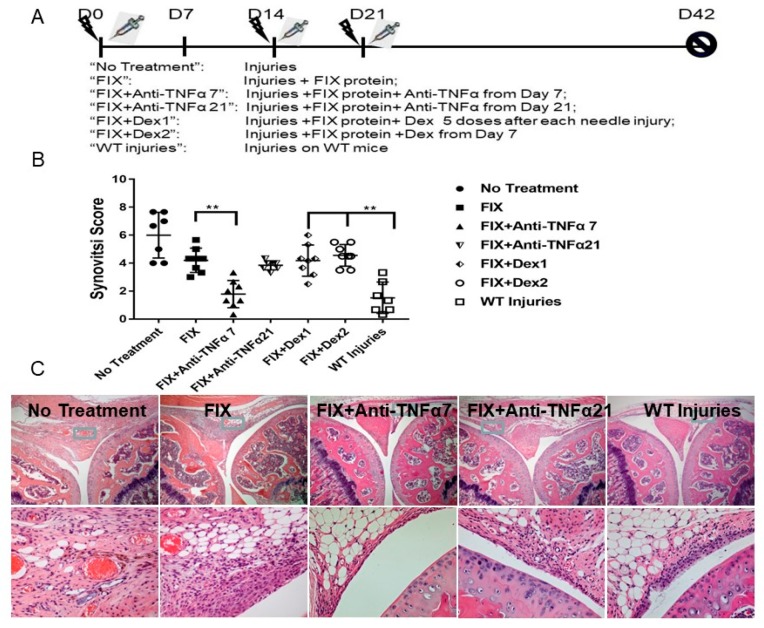
Efficacy of anti-TNFα in protecting against multiple bleeding-induced joint deterioration in FIX^−/−^ mice in vivo. (**A**): In vivo experimental design. “No Treatment”: Mice without any FIX protein. “On-demand”: FIX administered within fifteen minutes after each injury. “WT injuries”: WT mice subjected to the same injuries. 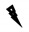 Represented injury; 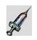 FIX protein treatment. (**B**): Synovitis score based on the murine synovitis grading system (*n* ≥ 7/group). (**C**): Representative histopathological images are shown. ** *p* < 0.01.

Hemarthroses were induced on day 0, 14, and 21 in FIX^−/−^ mice by needle injury as shown in Figure 1A. Groups of mice were listed below:

“No Treatment”: needle injury only;
“FIX”: FIX protein was administered after each needle injury;
“FIX + Anti-TNFα 7”: Besides FIX protein, anti-TNFα treatment was initiated from day 7;
“FIX + Anti-TNFα 21”: Anti-TNFα treatment was initiated from day 21;
“FIX + Dex1”: Besides FIX protein after each needle injury, Dex was administered for 5 consecutive days after each needle injury;
“FIX + Dex2”: Dex was administrated on day 7 every other day for a total 10 doses, a schedule identical to the “FIX + Anti-TNFα7” group;
“WT injuries”: Hemostatically normal mice subjected to the same injuries and sacrificed at week 6 to serve as the control group.

The doses for drugs were: FIX protein, 175 IU/kg intravenously; anti-TNFα 5 mg/kg per dose subcutaneously (s.c.); dexamethasone, 0.6 mg/kg intravenously.

All mice treated as described above were sacrificed at week 6.

#### 2.1.3. Histologic Grading

Hemophilic synovitis in injured and uninjured joints was graded according to a validated system [14,15], based on synovial hyperplasia (0–3 points), vascularity (0–3 points), and the presence of discoloration, blood, villi, or cartilage erosion (0 or 1 point for each), resulting in a combined score of 0–10 points for increasing pathology. Modified Mankin’s score was also employed based on hematoxylin and eosin (H&E) and Safranin-O staining to grade the cartilage changes. Images were captured with a DMX-1200 color camera (Nikon, Melville, NY, USA). using the Act-1 software (Nikon, Melville, NY, USA).

#### 2.1.4. TNFα in Synovial Fluid and Multiplex Cytokine Measurement

Synovial lavage was collected as previously described by washing out the synovial fluid twice with 25 μL normal saline. TNFα from synovial lavage and plasma was measured on a Bio-Plex 200 system (Bio-Rad, Hercules, CA, USA) using FMAP reagents from R&D Systems (Minneapolis, MN, USA) according to their instructions. Curve-fitting for reporting the primary concentration data was performed with the onboard Bio-Plex Manager v.5.0 software.

#### 2.1.5. Macrophage Immunostaining

The collection, handling, and processing of knee joint tissues were performed as previously described [8,13]. Macrophages were recognized by immunostaining with rat antibody against mouse macrophage specific F4/80 antigen (Serotec, Raleigh, NC, USA), with biotinylated anti–rat IgG (Vector Labs, Burlingame, CA, USA) as the secondary antibody [8,16]. Quantitative analysis were performed by counting of cells with positive staining in synovium.

### 2.2. Patient Study

#### 2.2.1. Patient Recruitment

**Figure 2:**
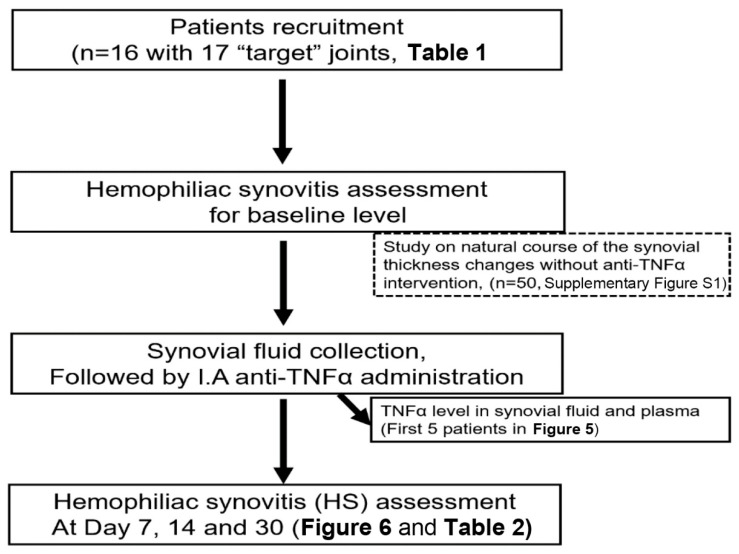
The consort diagram for patients study. I.A, intra-articular.

This was a single-center observational study conducted in the Clinical Medical College, Yangzhou University, China. An approval was obtained from the Ethical Review Board of the affiliated hospital. After obtaining their written informed consent, sixteen hemophilia patients (12 for Hemophilia A and 4 for hemophilia B) with a total of seventeen target joints were recruited in this study. Inclusion criteria were patients with “target” joint(s) in which three or more spontaneous bleeds have occurred within a consecutive 6-month period defined by WFH guideline [17] and ultrasound confirmation of the persistence of chronic synovitis [18] with frequent, recurrent bleeding that is not controlled by other means. The exclusion criterion was patients with an active infection, such as tuberculosis, HIV, hepatitis, and sepsis, cancer and cardiovascular diseases. The demographic characteristics are summarized in Table 1 and the design for patients study was displayed in Figure 2.

In order to observe the natural course of the synovial thickness changes without anti-TNFα therapy in hemophilia patients with “target” joints, a total of 50 joints from 14 patients, either on low dose prophylaxis (11/14, FVIII concentrates 8–12 IU/kg, 2–3 times per week) or on-demand therapy (3/14), were retrospectively reviewed. Joints were monitored by ultrasound in a period of 3 to 12 months (Supplementary Materials Figure S1).

#### 2.2.2. Synovial Fluid Harvesting and Anti-TNFα Administration from Patients with Hemophilia Arthropathy

Synovial fluid were collected under anesthesia via joint aspiration with an 18 G syringe from patients with joint effusion just before intra-articular injection [19]. Venous blood (2 mL) was directly withdrawn by a venipuncture into vacutainer tubes (Becton-Dickinson) from all participants at each visit before synovial fluid collection. 15–20 IU/kg Factor VIII concentrate (recombinant FVIII or plasma derived FVIII) was administered before joint aspiration and 12 h post-procedure. Another dose of FVIII concentrate was administered the following day according to the WFH guideline [17]. The dose for FIX was 30–40 IU/kg. Patients then returned to their previous treatment strategies.

After aspiration of the synovial fluid, a dose of anti-TNFα (etanercept, 25 mg per joint, a dose similar to that in RA patient as reported [20]) was directly injected into the joint space for patients receiving anti-TNFα therapy.

#### 2.2.3. TNFα Measurement in Plasma and Synovial Fluid

For human chemokine/cytokine measurement, TNF-α in a 45-cytokine panel was measured by Shanghai TissueBank Biotechnology Co. Ltd. (Shanghai, China) on a Luminex^®^100/200^TM^ (Luminex Corporation, Austin, TX, USA), equipped with xPONENT^®^ 3.1 software using custom kits (R&D systems, Shanghai, China). Cytokine levels were expressed in picograms per milliliter (pg/mL). Levels below the detection limit of each cytokine were defined as 0.

#### 2.2.4. Hemophiliac Synovitis (HS) Assessment and Monitoring

HS was assessed and monitored using B-mode and Power Doppler Ultrasound (PDUS) by two *sonologists* (Drs. Yang and Xia) with experience in musculoskeletal ultrasound. Ultrasonic examinations were performed with a real-time scanner (Philip EPIQ5, Ultrasound system, Royal Dutch Philips Electronics Ltd., Amsterdam, The Netherlands) equipped with a multi-frequency liner matrix array transducer (L5-12 MHz), using a widely described standardized scanning technique [21]. A modified Ultrasound scoring system [22,23] which was based on HEAD-US and incorporated with two parameters, joint effusion and synovial hypertrophy with angiogenesis from Melchiorre’s system [20] was used. All ultrasound examinations were carried out in a dark room with a stable temperature of 22 °C. Patients rested for at least 15 min before the ultrasound examination and were asked to avoid caffeine, tea, alcohol, sports, and smoking for 8 h until the examination.

The thickness of synovial membrane was measured (mm). Areas with thickness greater than 1.5 mm indicated synovial hypertrophy. When effusion was present, thickness of the synovial membrane was measured twice (i.e., in the swollen joint) and after pressure is applied with a transducer. To further monitor whether each location with affected joints had improvement, thickness of the synovial membrane was measured in multiple areas, especially in the zones of the suprapatellar and parapatellar recesses of the knee joint and the anterior and posterior recesses of the elbow and ankle. Two parameters were employed before and after treatment, (1): “synovial thickness in area with maximum change”, indicating the “biggest” changes among multiple measurements for certain area of synovium in the affected joint post IA injection and (2): “Mean synovial thickness of all evaluated areas”, representing the mean value from multiple measurements for all evaluated areas (Table 2).

PDUS was performed to detect synovial vascularity, which was defined as color-flow signals in structures between the capsule and bone surface [24]. The intra-articular PDUS signal was graded on a semi-quantitative scale system from zero to two: 0 = absence, no vessel signals; 1 = vessel signals in region of interest (ROI) < 3 flags; 2 = vessel signals in ROI > 3 flags or in more than half of the intra-articular area.

### 2.3. Statistical Analysis

Data are expressed as mean ± SEM. All data were analyzed by one-way analysis of variance and Tukey’s multiple comparison test in GraphPad Prism 7 for Windows (La Jolla, CA, USA). An adjusted *p* value of < 0.05 was considered statistically significant.

## 3. Result

### 3.1. Hemarthroses Elevated TNFα while Anti-TNFα Decreased TNFα Production in Synovial Fluid

**Figure 3:**
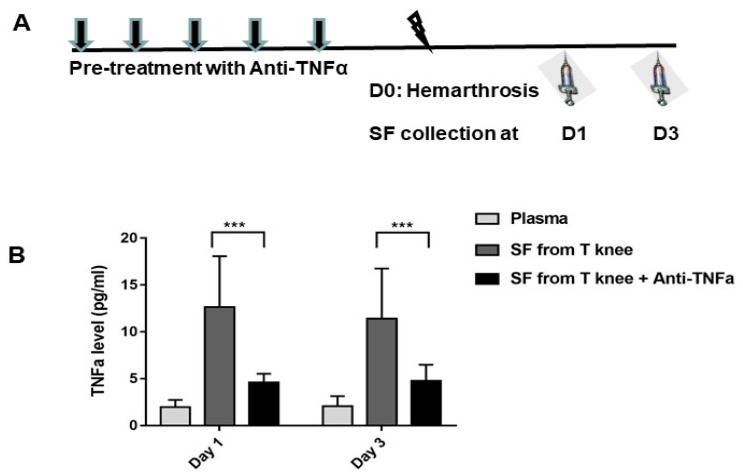
Hemarthrosis elevated TNFα while anti-TNFα decreased TNFα production in the synovial fluid. (**A**): FIX^−/−^ mice were pre-treated with 5 doses of daily anti-TNFα of etanercept 5 mg/kg s.c.; normal saline was administered to the control. On day 0, pretreated mice were subjected to hemarthrosis induction (arrow head). Synovial lavage from injured knee (“SF from T knee,” “SF from T knee + TNFi”) and contralateral knee were collected on days 1 and 3. (**B**): TNFα levels were detected. Results are presented as mean ± standard deviation. Synovial lavage from contralateral knee was undetectable on days 1 and 3. *N* = 7–8 for each time point. *** *p* < 0.001. Each value represents the mean ± standard deviation.

First, to determine whether hemarthrosis can induce the secretion of the pro-inflammatory cytokine, TNFα, in synovial fluid, hemarthrosis was induced in FIX^−/−^ mice and synovial lavage was collected on days 1 and 3. TNFα was undetectable in synovial lavage from uninjured FIX^−/−^ mice (the limit of detection reported as < 0.5 pg/mL). Nonetheless, joint hemorrhage led to a significant elevation in TNFα level in synovial lavage (12.6 ± 5.4 and 11.4 ± 5.3 pg/mL on days 1 and 3, respectively) as shown in Figure 3B. 

To investigate whether anti-TNFα can decrease TNFα yield in synovial fluid, FIX^−/−^ mice were pre-treated with 5 daily doses of anti-TNFα (5 mg/kg s.c) (Figure 3A). After hemarthrosis induction, synovial lavage was collected on days 1 and 3 (*n* = 7–8/time point) from the injured knee joint, with naïve joint as the baseline. Systemic administration of anti-TNFα decreased TNFα production in the synovial lavage (4.6 ± 0.9 and 4.7 ± 1.7 pg/mL on days 1 and 3, respectively. Both *p* < 0.01 compared with no anti-TNFα treatment controls) following hemarthrosis comparison to treatment without anti-TNFα.

### 3.2. Anti-TNFα Decreased Joint Deterioration after Multiple Intraarticular Hemorrhage in FIX^−/−^ Mice

To remodel a scenario where limited or multiple hemarthroses occurred before a novel therapy option, in FIX^−/−^ mice, a schedule of anti-TNFα co-therapy was selected for modeling either after one episode of joint hemorrhage (day 7 in this study, to resemble a condition with pre-existing joint damage after a few bleeding episodes) or after multiple episodes of joint bleeding (day 21) where three hemarthroses episodes already occurred, to mimic a condition resembling the occurrence of multiple bleeding episodes before any novel therapy (Figure 1A). In a separate group of animals, we could establish the development of synovitis on day 7 after one injury with I.V FIX (175 IU/kg) treatment (synovitis score of 3.1/10, data not shown).

Without any FIX coverage in FIX^−/−^ mice, about 40% animals survived the three recurrent hemarthroses induction, whereas most of the animals survived with FIX treatment. A significant pathology change developed and graded as synovitis score of 6.0 ± 1.6. Without anti-TNFα co-therapy, hemostasis support by “on-demand” FIX led to a pathology score of 4.2 ± 0.8 (Figure 1B and representative histology as shown in Figure 1C). However, co-administration of anti-TNFα on day 7 led to improved pathology changes (synovitis score 1.8 ± 0.9, *p* < 0.01 for “FIX” vs. “FIX + Anti-TNFα 7”). The mild change did not differ from that observed in wild type mice (“WT Injuries”) that experienced the same injuries (1.5 ± 1.1, *p* = 0.99 for “FIX + Anti-TNFα 7” vs. “WT injuries”). Nonetheless, if anti-TNFα therapy was initiated after multiple bleeding episodes (day 21 after three injuries), no additional benefit was achieved (Synovitis score of 3.8 ± 0.3, a score with no statistical difference compared to FIX-only “on-demand” therapy “FIX” group. Consistent to our previous study using anti-IL-6 co-therapy [8], cartilage changes were not dramatic when Mankin’s score system was adopted (data not shown).

Given Dexamethasone was also employed for hemophilia arthropathy management in clinical setting, in our animal study, two regimes were designed, either after each hemarthroses induction for 5 consecutive days, or from day 7 and administered every other day for a total 10 doses to compare the group of “FIX + Anti-TNFα7” side by side. As displayed in Figure 1, only a slight protection was seen in “FIX + Dex 1” compared to “no treatment” group, when dexamethasone treatment began immediately after each hemarthrosis induction. However, no regimen with dexamethasone resulted in any additional protection compared to FIX-only treatment.

### 3.3. Anti-TNFα Decreased Macrophage Infiltration or Proliferation in the Synovium

**Figure 4:**
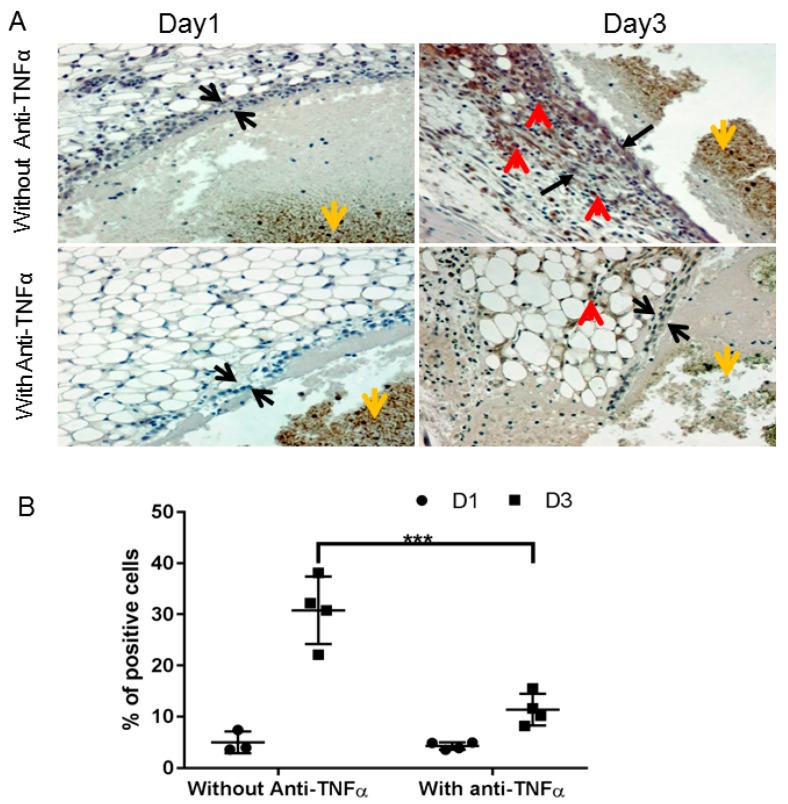
Anti-TNFα decreased macrophage infiltration/proliferation into the synovium. (**A**): FIX^−/−^ mice were pre-treated with 5 doses of etanercept 5 mg/kg s.c., as described in Figure 1A (“With Anti-TNFα”); mice in control group were treated with normal saline (“Without Anti-TNFα”). Hemarthrosis was induced by needle injury on the left knee joint. Days 1 and 3 after hemarthrosis induction, the treated knee joint was collected for macrophage immunochemistry staining as described in Methods. Black arrow represents the synovial lining; red arrow depicts positive macrophage staining; and yellow arrow represents the flesh blood hemorrhage in the joint space. (**B**): Quantitative analysis, percentage of positively stained cells, were performed by counting of cells in synovium. *** *p* < 0.01.

Abnormal wound healing in the hemophilic joint is characterized by prolonged and pathogenic residence of these inflammatory cells and the pro-inflammatory cytokines that they produce [14,25]. Hence, we investigated if anti-TNFα administration can affect the influx of monocyte/macrophage into the synovium after bleeding challenge. As shown in Figure 4A, almost no macrophage positive staining was observed in the synovium with or without anti-TNFα treatment on day 1. However, a significantly different pattern was observed with or without anti-TNFα therapy on day 3, as quantified in Figure 4B. We also observed strong macrophage staining in the tissues of animals treated only with the clotting factor, which was minimized with co-administration of anti-TNFα. This suggests that anti-TNFα might decrease monocyte/macrophage infiltration or proliferation in the synovium during hemarthroses.

### 3.4. Elevated TNFα in the Synovial Fluid of Patients with Hemophilia

In the patients study, a total of 16 patients with hemophilia (PWH) with 17 “target” joints were recruited (Table 1 and Figure 2). Synovial fluid and plasma were collected from the first recruited 5 patients and 45-panels of cytokines/chemokines, including TNFα, were measured. 

**Figure 5:**
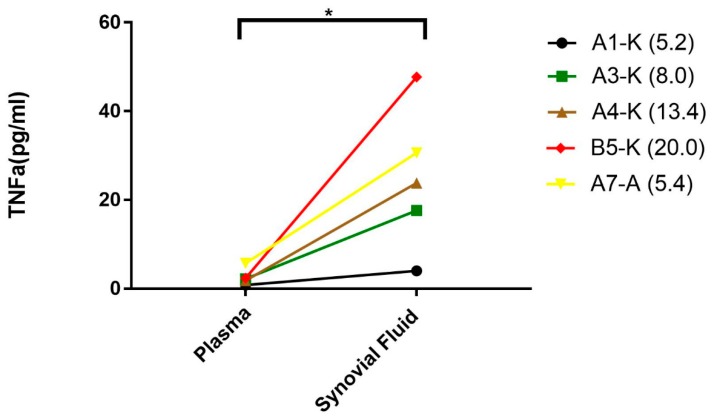
Elevated levels of TNFα in the synovial fluid of patients with hemophilic arthropathy. Plasma and synovial fluid were collected from hemophilia patients with hemophilic arthropathy to measure TNFα levels. Values in parentheses represented the ratios of TNFα level between the synovial fluid and plasma. * *p* < 0.05.

TNFα levels in synovial fluid were 5.2–20-folds higher than in plasma (*p* < 0.05) in PWH (Figure 5). To confirm the increased cytokines due to the synovial inflammation instead of passively being brought into the joint space by the hemarthroses breakthrough, hemoglobin from the synovial was also measured as a reference [26] (data not shown). About 3–4 folds lower hemoglobin (26–40 g/L vs. 140 g/L in whole blood) has been observed which suggested that elevated cytokines in the synovial fluid attributed to de novo production after hemarthroses.

### 3.5. Intra-Articular Administration of Anti-TNFα Decreased Synovial Thickness and Vascularity in Patients with HA

Intra-articular administration of tumor necrosis factor α (TNFα) inhibitor has been shown effective for the treatment of rheumatoid arthritis [20,27] with potential less systemic adverse effects of anti-TNFα [11]. To extend our in vivo finding in the hemophilia mouse model, a proof of concept study was performed where anti-TNFα was administered directly intra-articularly.

After aspiration of the synovial fluid, a dose of etanercept (25 mg per joint) was directly injected into the joint space. As shown in Table 2, the synovial thickness of area with maximum decrease and the percentage decreases, the mean synovial thickness of all evaluated areas and the ranges were recorded for each patient. All the 17 target joints displayed responses to anti-TNFα with a different degree. Maximum decrease (a range of 11–64% decrease on D7, 14–67% on D14 and 27–69% on D30) in synovial thickness and Mean decrease in synovial thickness were consistently observed on day 7 to day 30 after intra-articularly anti-TNFα injection. The statistical significances (*p* < 0.01) existed among any time points (day 7, 14 and 30, except for “mean decrease” at day 7) compared to baseline before treatment.

In the control group without anti-TNFα treatment, during a period of 3 to 12 months follow-up, only a slight decrease of synovial thickness was observed in 3 joints (6%, 3/50) in two patients (from 8.4 mm to 7.1 mm in right knee for Patient C5, 9 mm to 8.9 mm in right elbow, and 10.4 mm to 10.1 mm in left elbow of Patient C3, both with a 12 months interval) as demonstrated in Supplementary Materials Figure S1. The results suggested that spontaneous decrease of synovial membrane thickness may rarely occur in the majority of PWHs without effective therapeutic intervention.

**Figure 6:**
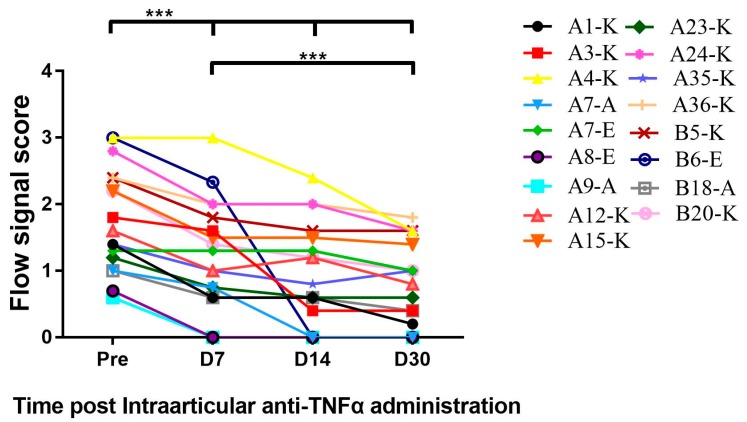
Intra-articular (I.A) administration of the TNFα antagonist decreased synovial vascularity. Synovial vascularity was detected by Power Doppler Ultrasound. As described in Section 2.2, colorful blood flow signals which were found between the capsule and bone surface inside the structures were observed decreased on days 7, 14 and 30 post-I.A administration in comparison to signals pre-treatment, respectively. *** *p* < 0.001.

Synovial vascularity, which represented neo-angiogenesis or inflammation, has been detected by blood flow signal using the ultrasonic system. As shown in Figure 6, all patients had decreased synovial vascularity in the “target” joints during the follow-up period from day 7 to day 30 compared to pre-injection after anti-TNFα administration with statistical significance at each time point. Future study will demonstrate the length of time that such benefit can persist or whether repeated administration will further improve the efficacy.

## 4. Discussion

Pro-inflammatory cytokines have been reported to be involved in the pathogenesis of synovitis [25,28] as interactions within the cytokine network and other inflammatory mediators in the affected joint can create an inflammatory environment. Theoretically, an anti-cytokine approach can be used to protect against bleeding in damaged joints; however, limited studies have shown that intervention of the cytokine network may serve as a novel therapeutic direction [5,29,30,31]; this includes our previous study with anti-IL-6 in hemophilia A mice [8].

As the management of HA has become a major concern, especially in undeveloped countries [1,2], a novel adjuvant therapy for hemophilic arthropathy management is necessary. Current prophylactic regimens do not fully prevent joint bleeding; hence, some patients can still develop joint disease [3,4].

Given the similar pathology changes between hemophilia A and B mouse model by our study [8] and publications [7,9,12,32], we assumed that there should be no difference of response to anti-TNFα between hemophilia A and B patients. Therefore, patients with either hemophilia A or B patients were recruited in this pilot study. Indeed, no difference of the response to anti-TNF therapy was observed between hemophilia A and B patients.

In the present study, elevated TNFα in the synovial lavage was identified in a hemophilia mouse model and patients with hemophilic arthropathy. Furthermore, we found that anti-TNFα, when initiated prior to the development of longstanding synovitis, can prevent hemophilic arthropathy caused by repeated intra-articular hemorrhage in hemophilia B mouse model. This occurs via a mechanism that can be attributed to a decrease in macrophage infiltration/proliferation into the synovium. Besides the efficacy to augment hemophilic arthropathy management, we also found that co-therapy with anti-TNFα minimized the immune response against infused clotting factor to decrease the risk of anti-FIX inhibitor development; this can also be related to the maintenance of homeostasis of Tregs after anti-TNFα administration. Interestingly, the in vivo finding demonstrated significant translational significance. A single dose of anti-TNFα via intra-articular administration decreased synovial thickness and the synovial vascularity observed on days 7 to 30 in the “target” joints of PWH.

Among the pro-inflammatory cytokines, TNFα has a broad spectrum of effects on the inflammatory process and can regulate IL-1 synthesis for the induction of IL-6 production [33]. Properties of TNFα, including the upregulation of adhesion molecules on endothelial cells, increase the migration of cells into inflamed joints. Consequently, TNF antagonists, e.g., etanercept, are clinically efficacious for the treatment of RA as they delay joint destruction via several mechanisms, such as decreasing cell recruitment to the inflamed joint [34,35] and synovial chemotactic cytokine expression [36]. In a recent study, Maneti M et al. reported the crucial role of TNF-α/TNF-R system in the pathogenesis of hemophilic athropathy, therefore can be employed as a new attractive target for the prevention and treatment of joint damage in hemophilic arthropathy patients [37], which was proven by the current study.

Maintaining the vascular integrity is critical to the prevention of bleeding in hemophilia. The network of multiple signaling pathways and molecules, including VEGF and platelet-derived growth factor (PDGF), plays a significant role in regulating angiogenesis, through pericyte recruitment and stabilization of neovessels [38]. In a non-hemophilia setting, TNF-α and VEGF-A have been reported to be associated with pathological angiogenesis, with disrupted vascular integrity, vascular leakage, and infiltration of inflammatory cells. In addition, a protein that blocks these cytokines could concomitantly reduce abnormal vascular tufts and the number of F4/80(+) macrophages in a retinopathy model [39].

The role of inflammation or neo-angiogenesis in the pathogenesis [7,28,32,40,41] of hemophilic arthropathy has been extensively studied using mouse models by our research team [14] and others. The increase in multiple pro-inflammatory cytokines/chemokines in patients with hemophilic arthropathy in this study supports the notion that pro-inflammatory cytokines/chemokines are attributed to the pathogenesis of hemophilic arthropathy. In hemophilia A rats, hemarthroses resulted in rapid cartilage and bone damage and the onset of synovial inflammation [42]. Nonetheless, modulating cytokine approaches, e.g., IL-6 receptor antagonist [8] and the anti-inflammatory cytokines of IL-4/IL-10 [30,43,44,45], can help to protect against joint damage after hemarthroses breakthrough.

There is only limited clinical data to support the administration of anti-TNFα for hemophilia management. Nonetheless, Melchiorre et al. demonstrated that using anti-TNFα [46] therapy to treat concurrent autoimmune disease (RA or psoriasis) symptoms in three patients with underlying hemophilic arthropathy decreased synovitis and greatly decreased the frequency of hemarthroses. Our study further expands on the feasibility of using TNFα blockade for hemophilic arthropathy management.

Unlike our findings, in an in vitro study using human cartilage culture, blocking IL-1β protected blood-induced cartilage damage. However, blocking TNFα did not display any benefit. We attributed these discrepancies partially to the difference in responses between the techniques (i.e., in vivo vs. in vitro). TNFα might play more critical roles in driving synovial inflammation instead of direct cartilage destruction [10]. Nevertheless, our results suggest that early initiation of anti-TNFα therapy rather than after multiple bleeding episodes is needed. Given the benefit of direct intra-articular corticosteroid administration was reported to be a decrease in pain in patients with chronic hemophilic arthropathy. A placebo-controlled, randomized trial (performed when the supply of clotting factor concentrates were less secure) showed that a 5-day course of oral corticosteroid after acute hemarthroses resulted in a lower requirement of replacement clotting factor to promote a return to normal joint function [47]. In this report, co-administration of dexamethasone either with the 5-day course after each hemarthrosis or a regime resembling the anti-TNFα starting from day 7 resulted in no additional protection compared to FIX-only treatment.

Another interesting observation is that repeated exposure to FIX during the bleeding episodes led to an increase in inhibitor formation of treated animals, and this risk was minimized with anti-TNFα co-therapy (Supplemental Table S1). The outcome can be attributed to preventing the decrease in Tregs during an immune response after anti-TNFα treatment (Supplementary Materials Figure S2). We proposed that this could be attributed to the inflammatory environment evoked by the multiple intra-articular hemorrhages where “danger signals” were released [48] which may assist FIX presentation to T cells. Given the less risk of inhibitor in hemophilia B setting, whether this observation can be extended to hemophilia A management warrants further investigation. 

It is important that we highlight that this is merely a proof of concept study; hence, continuous work is still needed to demonstrate the benefit and dose optimization of the TNFα antagonist before it can be fully translated for hemophilic arthropathy management. Given the multiple cytokine changes that can be related to hemophilic arthropathy, whether adopting multiple anti-cytokine approaches can be more efficacious than conventional approaches is currently being tested in an ongoing study. With half-life increased clotting factors available, whether addition of TNF blockade improve better wound healing, esp. for already developed target joint, warrants further investigation.

In summary, the results obtained from the hemophilia mouse model and patients with hemophilic arthropathy show that TNFα is associated with the pathogenicity of hemophilic arthropathy. Administering anti-TNFα as an adjuvant therapy could improve the outcome of HA and aid in its management.

## Figures and Tables

**Table 1:** Demographic data of patients.

8	Age (Year)	Type	FVIII:C/IX	Target Joint/Nomenclature	AJBR 6 Months Before I.A Injection	Treatment
			%		Times	
A1	30	HA	<1%	RK (A1-K)	8	OD
A3	20	HA	<1%	RK (A3-K)	11	OD
A4	28	HA	<1%	LK (A4-K)	15	OD
A7	22	HA	1%	LA (A7-A)	10	OD
				RE (A7-E)	8	
A8	13	HA	<1%	RE (A8-E)	7	Pro (600U, 3/week)
A9	16	HA	<1%	RA (A9-A)	6	OD
A12	26	HA	1%	LK (A12-K)	20	OD
A15	25	HA	<1%	LK (A15-K)	18	Pro (400U 2/week)
A23	18	HA	1.1%	RK (A23-K)	10	Pro (1000U, 3/week)
A24	16	HA	<1%	LK (A24-K)	16	OD
A35	38	HA	<1%	LK (A35-K)	8	OD
A36	43	HA	<1%	LK (A36-K)	12	OD
B5	24	HB	<1%	LK (B5-K)	13	OD
B6	30	HB	<1%	LE (B6-E)	9	OD
B18	22	HB	1.5%	LA (B18-A)	4	Pro (PCC 1000IU, q5d)
B20	25	HB	<1%	RK (B20-K)	8	OD

HA: Hemophilia A; HB: Hemophilia B; R: Right; L: Left; K: Knee; A: Ankle; E: Elbow; OD: on-demand; Pro: prophylaxis; I.A: Intra-articular administration. PCC: Prothrombin Concentrates Complex.

**Table 2:** The dynamic changes in thickness of the synovial membrane after I.A administration of Anti-TNFα.

Target Joint ID	Synovial Thickness in Area with Maximum Change (mm (−%))				Mean Synovial Thickness of All Evaluated Areas (mm)				Range of Synovial Thickness in All Evaluated Areas (mm)			
	Pre-	D7	D14	D30	Pre-	D7	D14	D30	Pre-	D7	D14	D30
A1-K	11	3.9 (−64.5)	3.6 (−67.3)	3.4 (−69.1)	6.06	4.16	4.06	3.72	4.2–11.0	2.5–6.1	2.1–5.2	3.4–4.2
A3-K	4.9	3.5 (−28.6)	2.1 (−57.0)	1.5 (−69.4)	5.04	4.14	3.68	3.36	3.6–7.8	3.1–5.8	2.1–5.2	1.5–5.0
A4-K	25	16 (−36.0)	12 (−52.0)	10 (−60.0)	14.66	11.04	8.12	8.08	7.8–25	6.6–16.0	4.6–14.0	3.7–10.4
A7-A	8.5	5.5 (−35.3)	4.9 (−42.4)	4.9 (−42.4)	6.35	5.57	4.9	4.13	3.7–8.5	3.6–7.9	3.2–6.2	3.0–4.9
A7-E	18.3	15.4 (−15.8)	8.4 (−54.1)	8 (−56.3)	8.8	7.9	6.1	6	3.8–18.3	3.5–15.4	3.4–8.4	3.4–8.0
A8-E	7.5	4.5 (−40.0)	3.7 (−50.7)	3.7 (−50.7)	9	6.43	4.73	4.7	6.4–13.1	4.1–10.7	3.7–6.7	3.7–6.5
A9-A	13	NA (NA)	10.7 (−17.7)	8.8 (−32.3)	8.13	NA	5.06	7.56	4.6–13.0	NA	2.8–10.7	3.9–10.0
A12-K	11	6.8 (−38.2)	7.2 (−34.5)	7.4 (−32.7)	7.78	6.5	5.8	6.38	5.0–11.0	4.7–10.3	4.8–11.2	4.4–8.4
A15-K	16.2	14.2 (−12.3)	9.4 (−42.0)	10.8 (−33.3)	13.24	11.2	9.8	10.14	9.9–16.2	6.4–15.8	5.9–14.8	7.5–11.0
A23-K	16.9	7.4 (−56.2)	7.8 (−53.8)	5.8 (−65.7)	12.1	8.4	9.36	7.8	7.3–16.9	6.7–15.4	6.4–12.9	5.5–10.8
A24-K	12	10.4 (−13.3)	5.9 (−50.8)	6.5 (−45.8)	12.82	9.4	7.9	8.38	11.9–13.8	6.6–13.2	5.9–13.5	6.5–10.2
A35-K	10.3	7.9 (−23.3)	7.1 (−31.1)	7.5 (−27.2)	9.88	8.68	8.04	8.32	6.2–12.7	4.7–10.8	4.9–11.2	5–10.9
A36-K	13	6.9 (−46.9)	6.4 (−50.8)	7.4 (−43.1)	9.3	8.4	8.2	8	5.5–13	5.1–10.5	4.9–11.1	5.5–10.2
B5-K	17.1	9.1 (−46.8)	6.1 (−64.3)	5.8 (−66.1)	16.74	12.7	9.6	8.68	13.5–18	9.1–16.9	6.0–15.7	5.8–11.8
B6-E	19.2	12.5 (−34.9)	8.8 (−54.2)	9.7 (−49.5)	15	12	10.3	10.4	8.8–19.2	9.5–14.0	8.3–13.8	8.3–13.0
B18-A	6.4	5.7 (−10.9)	5.5 (−14.1)	3.7 (−42.2)	5.23	NA	4.3	3.46	4.6–6.4	NA	3.4–5.0	3.2–3.7
B20-K	14.4	10.4 (−27.8)	6.7 (−53.5)	6.4 (−55.6)	12.4	12.2	8.9	8.4	7.9–16.4	7.5–17.6	7.9–13.4	8.4–14.1

“Synovial thickness (mm) in area with maximum change” displayed in the left panel. Values in parentheses represented the percentage decrease compared to thickness pre-treatment for each patient. “NA”: No data collected. For “Synovial thickness (mm) in the area with maximum change”, *p* all < 0.001 for comparisons: “d7” vs. “pre”, “d14” vs. “pre”, and “d30” vs. “pre”. For “Mean synovial thickness of all evaluated areas”, *p* = 0.39 for “d7” vs. “pre”; *p* < 0.01 for “d14” vs. “pre”; *p* < 0.01 for “d30” vs. “pre-”.

## Supplementary Materials

The following are available online at https://www.mdpi.com/2077-0383/9/1/75/s1.
