# Supplementary material for: A Translational Study of TNF-Alpha Antagonists as an Adjunctive Therapy for Preventing Hemophilic Arthropathy
Source: J Clin Med. 2019 Dec 27;9(1):75. doi: 10.3390/jcm9010075 (PMC7019955; doi:10.3390/jcm9010075)
Supplement: Supplementary file 1 [file jcm-09-00075-s001.pdf]

Journal of
Clinical Medicine

MDPI

A Translational Study of TNF-alpha Antagonists as an Adjunctive Therapy for Preventing Hemophilic Arthropathy

Feixu Zhang [1,2,†], Mengyang Xu [3,†], Qin Yang [3,†], Baolai Hua [3,*], Binglan Xia [3], Zhenyang Lin [2], Xiao Xiao [2], Paul E. Monahan [4,5,6] and Junjiang Sun [4,7,*]

Supplemental Materials and Methods:

FIX functional activity and anti-FIX Bethesda inhibitor assay

One-stage FIX activity assay (FIX-specific aPTT) and FIX Bethesda inhibitor assay were performed as described previously [1,2], using a START 4 Coagulation Analyzer (Diagnostica Stago, Asnières, France).

Flow cytometry

Freshly-isolated peripheral red blood cells-lysed cells were stained with APC-Cy7-conjugated anti-mouse CD4 antibody and FITC-conjugated CD25 antibody (BD Biosciences, San Jose, CA, USA) as described previously [3].

Statistics

Data are expressed as mean ± SEM. All data were analyzed by one-way analysis of variance and Tukey's multiple comparison test in GraphPad Prism 7 for Windows (La Jolla, CA, USA). An adjusted p value of < 0.05 was considered statistically significant. The chi square test was used for the analysis of inhibitor development.

Results

Anti-TNFα decreased the risk of anti-FIX inhibitor formation associated with FIX treatment in the joint hemorrhage in FIX$^{-/-}$ mice

FIX$^{-/-}$ mice developed FIX neutralizing antibodies (inhibitors) much less readily when exposed to the human FIX protein. As shown in Supplementary Table S1, when administered three doses of FIX treatment without bleeding induction ("Injuries only"), no mice developed FIX inhibition. Interestingly, repeated exposure to FIX during the bleeding episodes led to an increase in inhibitor formation in ~30.7% (12/39 in "Injuries + FIX") of treated animals. Strikingly, this risk of inhibitor formation was significantly decreased with anti-TNFα co-therapy (only 1 of the 58 treated animals, p < 0.001). Although the adjunctive dexamethasone did not result in any further protection against joint damage, the adjunctive corticosteroid was also associated with a lower incidence of inhibitor formation (10% of 3/30 mice in the adjunctive dexamethasone group) compared to 12 of 39 mice that received factor IX, without the anti-inflammatory factor, for bleeding (30.7%, p = 0.038 by chi-square test, Supplemental Table S1).

To further clarify whether immune tolerance was established after anti-TNFα treatment in mice, we extended the follow-up period to 16 weeks instead of 6 weeks as displayed in Figure 2. Consistently, only 1 mouse developed the anti-FIX inhibitor following anti-TNFα treatment at 16 weeks; this was compared to mice (4 of 17; including 1 mouse who disappeared at W16) that did not receive anti-TNFα treatment. However, when the mice were re-challenged by joint bleeding injuries and FIX treatment at week 20 ("W20 + 1 Inj") and 24 ("W24 + 2 Inj"), the difference in inhibitor

incidence disappeared as shown in Supplemental Table S2, implicating that anti-TNFα therapy did not establish long term immune tolerance during the treatment period.

Supplemental Table S1. FIX inhibitor formation in FIX$^{-/-}$ mice subjected to multiple knee joint bleeding induction.

Group	Total *N*	*N* of Inhibitor Formation (%)	Inhibitor Titer (BU)	*p*
Injuries Only	23	0	NA	NA
FIX only	11	0	NA	NA
Injuries+FIX	39	12 (30.7%)	1.9 ± 1.3 (0.8–5.1) *	NA
Injuries+FIX+Anti-TNFα	58	1 (1.7%)	0.7	<0.01
Injuries+FIX+Dex	30	3 (10.0%	2.1 ± 1.3 (0.8–3.5) *	<0.05

* Calculated in mice with detectable FIX inhibitor; *p* vs. "Injuries + FIX.".

Supplemental Table S2. Long-term follow-up of FIX inhibitor formation in FIX$^{-/-}$ mice.

	Injuries + FIX			Injuries + FIX + Anti-TNFα		
**Time**	**Total *N***	**Anti-FIX (+)**	**Titer (BU/mL)**	**Total *N***	Anti-FIX (+)	Titer (BU/mL)
W6	17	4 (23.5%)	0.6–2.5 (1.1 ± 0.93)	16	1 (6.2%)	0.67
W16	17	3 (17.6%)	0.5–1.1 (0.8 ± 0.3)	16	1 (6.2%)	0.72
W20+1 Inj	15	6 (40%)	0.6–2.0 (1.1 ± 0.5)	16	5 (31.2%)	0.6–1.1 (0.8 ± 0.2)
W24+2 Inj	15	5 (33.3%)	0.7–1.1 (0.8 ± 0.1)	16	5 (31.2%)	0.7–1.0 (0.8 ± 0.3)

FIX$^{-/-}$ mice were subjected to three repeated hemarthroses induction as displayed in Figure 2A with or without anti-TNFα co-therapy. At week (W) 16, surviving mice were subjected to another hemarthroses induction with FIX therapy, but without anti-TNFα. After an additional 4 weeks ("W20 + 1 Inj"), level of FIX inhibitor was measured. Another group of surviving mice was subjected to the same treatment at W20 then W24 ("W24 + 2 Inj"); the level of FIX inhibitor was then measured.

Anti-TNFα delayed the decrease in regulatory T cells (Tregs) in mice prone to FIX inhibitor formation

Regulatory T cells play a critical role in immune tolerance in the treatment of hemophilia [4,5]. Therefore, we determined whether anti-TNFα co-administration can affect Treg quantity in FIX$^{-/-}$ mice treated with FIX after multiple joint bleeding episodes. As shown in Supplementary Figure S2, naïve mice and anti-TNFα-only treated mice ("anti-TNF only") had ~6% of circulating Tregs, while in the mice that developed FIX inhibition ("Injuries + FIX Ab (+)"), a decrease in Treg (4.3% ± 0.7%, $p < 0.01$) was observed. In contrast, mice that received the same treatment but did not develop any anti-FIX inhibitor ("Injuries + FIX Ab (−)") displayed Tregs (5.9% ± 1.7%) similar to the naïve control. Interestingly, mice treated with anti-TNFα had a normal amount of Tregs. This suggests that anti-TNFα might prevent the decrease in Tregs during an immune response.

In addition to providing hemostasis, countering the inevitable inflammation that follows bleeding may decrease synovitis. However, this approach has to be initiated prior to the development of the longstanding synovitis. In addition, anti-inflammatory co-therapy might decrease the risk of inhibitor formation.

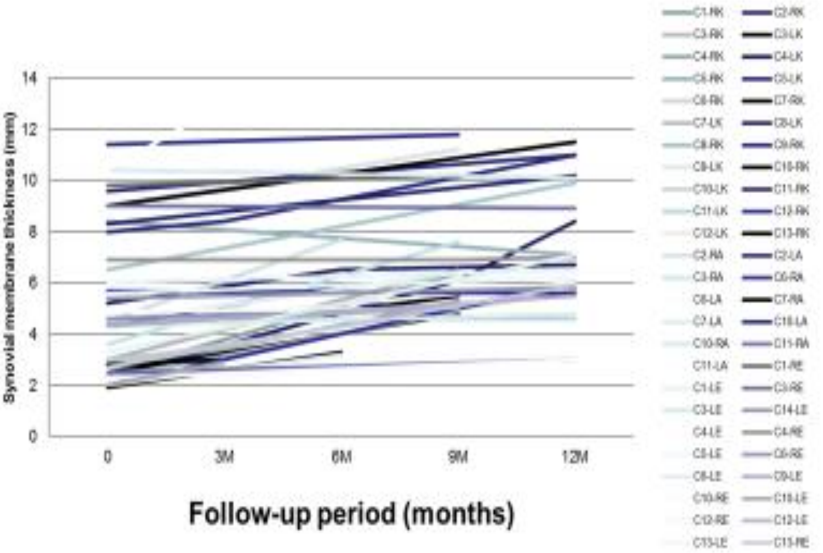

Supplementary Figure S1. Natural course of the synovial thickness changes in hemophilia patients with "target" joints. Each line represented one individual joint of the patient. R: Right; L: Left; K: Knee; A: Ankle; E: Elbow.

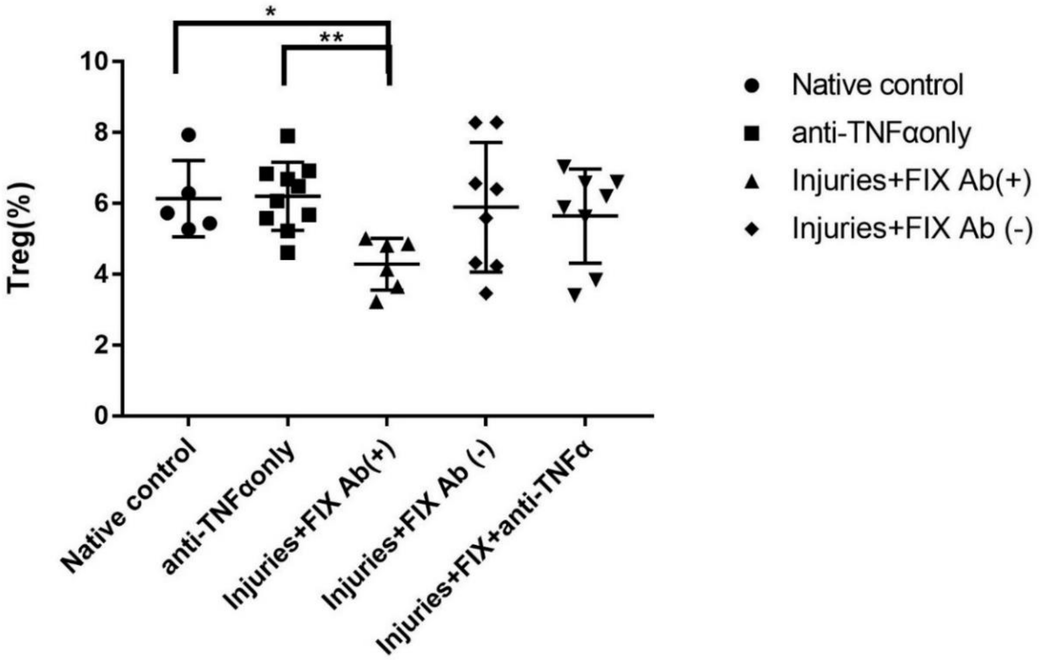

Supplementary Figure S2. Anti-TNFα prevented the decrease in regulatory T cells in mice with FIX inhibitor formation. $FIX^{-/-}$ mice were subjected to three hemarthroses induction with I.V FIX (175 IU/kg) after each bleeding episode as shown in Figure 2A, with or without anti-TNFα co-therapy. $CD4^{+}CD25^{+}$ regulatory T cells were detected by flow cytometry. Naïve control: naïve mouse without any treatment; Anti-TNFα only: mice administered only 10 doses of etanercept without any other treatment; "Injuries + FIX Ab (+)": mice that developed anti-FIX inhibitor after injuries and FIX treatment; "Injuries + FIX Ab (−)": mice that did not develop anti-FIX inhibitor after injuries and FIX treatment. * $p < 0.05$, ** $p < 0.01$.

References

1. Zhang, T.P.; Jin, D.Y.; Wardrop, R.M., III; Gui, T.; Maile, R.; Frelinger, J.A.; Stafford, D.W.; Monahan, P.E. Transgene expression levels and kinetics determine risk of humoral immune response modeled in factor IX knockout and missense mutant mice. *Gene Ther.* **2007**, *14*, 429–440.
2. Waters, B.; Qadura, M.; Burnett, E.; Chegeni, R.; Labelle, A.; Thompson, P.; Hough, C.; Lillicrap, D. Anti-CD3 prevents factor VIII inhibitor development in hemophilia A mice by a regulatory CD4+CD25+-dependent mechanism and by shifting cytokine production to favor a Th1 response. *Blood* **2009**, *113*, 193–203.
3. Hu, G.; Guo, D.; Key, N.S.; Conti-Fine, B.M. Cytokine production by CD4+ T cells specific for coagulation factor VIII in healthy subjects and haemophilia A patients. *Thromb. Haemost.* **2007**, *97*, 788–794.
4. Miao, C.H. Immunomodulation for inhibitors in hemophilia A: The important role of Treg cells. *Expert Rev. Hematol.* **2010**, *3*, 469–483.
5. Miao, C.H.; Harmeling, B.R.; Ziegler, S.F.; Yen, B.C.; Torgerson, T.; Chen, L.; Yau, R.J.; Peng, B.; Thompson, A.R.; Ochs, H.D.; et al. CD4+FOXP3+ regulatory T cells confer long-term regulation of factor VIII-specific immune responses in plasmid-mediated gene therapy-treated hemophilia mice. *Blood* **2009**, *114*, 4034–4044.
